# The effect of territorial awareness in a three-species cyclic predator–prey model

**DOI:** 10.1038/s41598-022-05845-0

**Published:** 2022-02-02

**Authors:** Xiaoyue Wang, Yikang Lu, Lei Shi, Junpyo Park

**Affiliations:** 1 School of Statistics and Mathematics, Yunnan University of Finance and Economics, Kunming, 650221 Yunnan China; 2 Department of Applied Mathematics, Kyung Hee University, Yongin, 17104 Republic of Korea

**Keywords:** Complex networks, Biodiversity

## Abstract

Recognizing territories is essential to decide behavior of population either human or animals, and interaction between groups or individuals according to the territorial awareness is universal. Understanding various mechanisms which affect on such species behaviors can be possible by evolutionary games, and in particular, the rock–paper–scissors (RPS) game has been played a key role as a paradigmatic model to explore biodiversity from microbiota to societies. Among paramount mechanisms in systems of RPS, the role of intraspecific interaction has been recently noted in terms of promoting biodiversity. Since intraspecific interaction is defined by an invasive reaction between individuals in the same group, the interaction may be also sensitive to the territorial awareness. To explore how territorial awareness-based intraspecific interaction can affect species biodiversity, we endow species with the mechanism in the classic RPS game. By means of the Monte-Carlo method, we find the phenomenon that the presence of species’ territorial awareness has an impact on intraspecific interaction which ultimately affects species biodiversity. At the same time, we also find that territorial awareness can play a significant role to the average waiting time for extinction which is numerically elucidated by exploiting the quantity: interface width statistic. Unlike prior research that concentrated solely on the relationship between interaction frequency and species diversity, our results shed lights on the important role of territorial awareness in models of RPS, and they reveal fascinating evolutionary outcomes in structured populations that are a unique consequence of such awareness behavior.

## Introduction

How to maintain biodiversity has always been one of fundamental and intensive issues in ecological sciences, and evolutionary games have been employed as useful tools to understand essential factors closely related to biodiversity^1–4^. In particular, for non-hierarchical, cyclic interaction among species that has been witnessed from experimental studies such as mating strategies among three side-blotched lizards in California^5^ and microbial populations^6–8^, the “rock–paper–scissors” (RPS) game has been adopted as a representative model to elucidate important mechanisms^9,10^. In this regard, over the past decade, evolutionary dynamics of cyclic interaction have been adopted extensively in both macroscopic and microscopic frameworks to elucidate rich phenomena in ecological sciences. While traditional approaches on RPS systems have been performed by employing mean-field systems macroscopically, the trend has been changed into incorporating mathematical theories and spatial domains after a milestone work^11^ that introduced the important role of individuals' mobility on biodiversity highlighted the importance of spatial mechanisms. Therefore, recent studies on RPS systems are conducted engaging with complex networks which show fast-growing as valuable tools to elucidate species biodiversity, and addressed significant roles of underlying mechanisms such as mutation^12^, evolution on continuous space^13^, sensitivity on initial species richness and spatial scales^14–18^, intraspecific interaction^15,19–22^, and effects of local environment^23–25^.

Among such great efforts on RPS games, some recent works have been focused on the role of intraspecific interaction, a form of interaction between individuals in the same species group that is quite common in ecological systems^26–28^, and found to be a necessary condition for species coexistence^29,30^. While the fundamental predator–prey relation is an interspecific interaction that is a natural mechanism in general ecosystems, intraspecific interaction is also a common feature that can occur during conflict on competing to possess essential resources between individuals. Microscopically, the exploitation of intraspecific interaction has been contributed to uncover a significant role to promote biodiversity, in particular species coexistence, on spatially extended systems. In contrast to the classic spatial RPS game^11^, recent studies on the RPS system with intraspecific interaction exhibit persistent coexistence when the strength of intraspecific interaction exceeds a certain value^15,18,19,21^. In addition, considering symmetry-breaking on intraspecific interaction can yield diverse survival states which can realize the survival of subgroup population^20,22^. For such features, we found that the effect of mobility has been disappeared, i.e., coexistence and diverse survival states are independent to mobility. In this regard, we may know that an imbalance of intraspecific interaction among groups can be closely related to species biodiversity.

In perspectives of ecological sciences, intraspecific interaction can occur with high likelihood when species undergo lack of essential resources, and unsatisfactory to environments may cause internal crack. Specifically, such a territorial behavior is universal and, as a device for leaving space for individuals in the available food supply and guiding the development of the species by limiting mating opportunities and protecting the young, it is an important factor in evolution of most species including humans^31–33^. Many mammalian carnivores that live in social groups defend their territory. In these carnivores, the territories of neighboring groups often overlap with little or no overlap, group members often use scent markers to mark territory boundaries, and native animals often engage in aggressive behavior against alien species found within their territory. For example, individuals within the same pride of African lions (${ Panthera\,leo }$) defend the territory together, and the territorial interaction between unrelated domains (i.e. the less related) will be the fiercest^34^; The dwarf mongoose’s territorial boundaries are closely linked to each other, with slightly overlapping areas, their groups are mainly made up of highly related individuals. When two groups of dwarf mongoose meet, they fight, while there is very little overt aggression among group members^35^; Spotted hyena ($Crocuta\,crocuta$) lives in social units or tribes whose members have established social relationships, i.e. they often engage in interactions that express mutual tolerance. But hyenas of different families are territorial^36,37^. Thus considering the territorial imbalance of (local) species is necessary to understand intraspecific interaction. Nevertheless, most results in previous works above have been established with constant rates: either uniform rate of intraspecific interaction for all groups^15,18,19,21^, or nonuniform rates depending on characteristic of groups^20,22^. In other words, given that intraspecific interaction is related to the territorial consciousness of individuals, it has not been rigorously studied. In this pursuit, as a new and neglected factor, we may wonder how individuals’ consciousness on land can affect individuals’ behavior, in particular intraspecific interaction. The main purpose of this paper is thus to demonstrate and establish the effect of “territorial awareness” (or territorial consciousness) on intraspecific interaction and its successive role on biodiversity in the RPS system in the individual level.

To search for a relation between territorial awareness and intraspecific interaction, we assume that the same species has two different attributes, represented by the distribution of individuals in two different territorial regions which will be realized by dividing two rectangles on the given square lattice, and intraspecific interaction will occur between individuals of the same species with different attributes. With these species are distributed on spatially extended systems, we take an adjustable parameter $k_{i}(i=A, B, C)$ to describe the sensitivity of intraspecific interaction to territorial awareness (or referred to as “sensitivity coefficient”) of species *i*. From Monte-Carlo simulations, we found that the existence of species’ territorial consciousness can hamper species diversity in general, causing the extinction of one attribute of the same species. But we also find that there is a general feature that the damage of territorial consciousness to species diversity slowed down with the increase of its own intensity when individuals' mobility is low.

## Results

### Model

**Figure 1:**
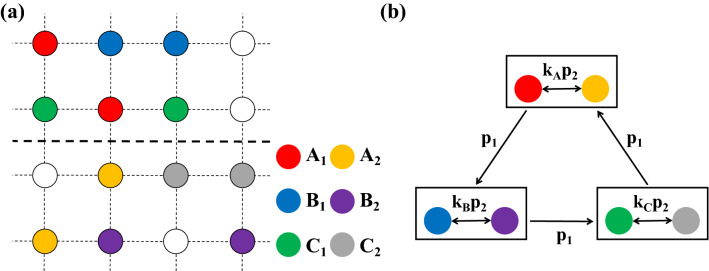
Schematic diagrams of network structure and the invasion rules among species. (**a**) Each circle represents a node, and individuals of species *A*, *B*, and *C* are evenly and randomly distributed on each node. To realize territorial awareness, the lattice is divided into two regions of equal size: the top and the bottom where the dashed line indicates the regional boundary. Two genera of the same species are distributed in different regions, and different color markers represent different species types. Nodes without color markers are empty nodes. (**b**) Interspecific interaction among three species *A*, *B*, and *C* (indicated by three boxes) occurs cyclically with a rate $p_1$. A box of each group describes the intraspecific interaction between individuals who belong to different territories where the interaction is regulated by territorial consciousness. Here intraspecific interaction in each group occurs with a rate $k_i \cdot p_2$ ($i \in \{A, B, C\}$).

To investigate the evolution of cyclically competing species with intraspecific interaction which sensitively plays to the territory awareness, we employ the spatial RPS model^11,19,20,23^. At the microscopic level, the model can be demonstrated on a lattice system, and for convenience, we consider a square lattice of size *N* with periodic boundary conditions where all sites have von Neumann neighbors. Each site can be occupied by an individual from one of the three species (referred to as *A*, *B*, and *C*, respectively) or left empty(*E*), and thus the system describes a limited carrying capacity. In addition, to explore the effect of territory awareness on intraspecific interaction, we assume that the given lattice is divided into two areas of the same size which may possibly realize different territorial ranges. Here we simply divide the two regions into the top and bottom halves of the given square lattice. To reflect the territorial awareness on intraspecific interaction, we distribute population in each group into two sub-networks randomly, and denote species $X_1$ for the top and $X_2$ for the bottom ($X \in \{A, B, C\}$) to distinguish the emergence of intraspecific interaction between individuals who lie on different domains. The distribution of all species with respect to the separation of the domain is illustrated in Fig. 1a.

Under the given assumption for the lattice, all the interactions between individuals occur within nearest neighboring sites by the following set of rules (see Fig. 1b):

1 $$\begin{aligned}&A_{i}+B_{j}\overset{p_{1}}{\longrightarrow }A_{i}+E,\quad B_{i}+C_{j}\overset{p_{1}}{\longrightarrow }B_{i}+E,\quad C_{i}+A_{j}\overset{p_{1}}{\longrightarrow }C_{i}+E, \end{aligned}$$

2 $$\begin{aligned}&A_{i}+A_{j}\overset{k_{A} \cdot p_{2}}{\longrightarrow }A_{i}+E\,\mathrm{or}\,E+A_{j}, \quad B_{i}+B_{j}\overset{k_{B} \cdot p_{2}}{\longrightarrow }B_{i}+E\,\mathrm{or}\,E+B_{j}, \quad C_{i}+C_{j}\overset{k_{C} \cdot p_{2}}{\longrightarrow }C_{i}+E\,\mathrm{or}\,E+C_{j},\quad i\ne j, \end{aligned}$$

3 $$\begin{aligned}&A_{i}+E\overset{r}{\longrightarrow }A_{i}+A_{i},\quad B_{i}+E\overset{r}{\longrightarrow }B_{i}+B_{i},\quad C_{i}+E\overset{r}{\longrightarrow }C_{i}+C_{i}, \end{aligned}$$

4 $$\begin{aligned}&A_{i}+\otimes \overset{m}{\longrightarrow }\otimes +A_{i},\quad B_{i}+\otimes \overset{m}{\longrightarrow }\otimes +B_{i},\quad C_{i}+\otimes \overset{m}{\longrightarrow }\otimes +C_{i}, \end{aligned}$$

where $i, j=1, 2$. The mark $\otimes$ stands for any species or empty sites. Relation (1) describes interspecific interaction among three species which occurs cyclically with a rate $p_1$: $A_{i}$ dominates $B_{i}$, $B_{i}$ dominates $C_{i}$, and $C_{i}$ dominates $A_{i}$ ($i=1, 2$). The defeated individual dies and the site becomes an empty site. Relation (2) demonstrates the intraspecific interaction which will sensitively depend on territorial awareness. Since we assume the intraspecific interaction is related to the territorial consciousness, the rate in each species may be defined by $k_{A} \cdot p_{2}$, $k_{B} \cdot p_{2}$, $k_{C} \cdot p_{2}$ for species *A*, *B*, *C*, respectively, where $p_{2}$ is the given rate of interaction, *k* is the the sensitive parameter to territorial awareness. Similar to previous works, the result of intraspecific interaction eventually results in a death of one individual at random with a 1/2 chance. Relation (3) stands for the reproduction with a rate *r* which is allowed when an empty site in neighbors is selected, and migration defined by an exchange between two neighboring sites is denoted in Relation (4). Based on the theory of random walks^38^, it occurs with a rate $m=2MN$ where *M* and *N* indicate individuals' mobility and a system size, respectively, as usual to previous works. Thus, an actual time step is defined when each individuals has interacted with others once on average, i.e., *N* pairwise interactions will occur in one actual time step unit. In order to make an unbiased comparison with previous works^15,19–21^ and for the convenience of interpretations, we assume parameters as $p_{1}=p_{2}=r=1$ and $k_{A}=k_{B}=k_{C}=k$ (see the Methods for the meanings of specific parameters) in our simulations. Three species are divided into two types to distinguish distributions on different regions: $A_{1,2}$, $B_{1,2}$, and $C_{1,2}$, and randomly distributed initially on a square lattice of size $N= 300 \times 300$. In addition, in all our simulations, species coexistence refers to the coexistence of $A_i$, $B_j$, and $C_k$ for any combination of $i,\,j,\,k \in \left\{ 1,\,2 \right\}$.

### Biodiversity under territorial awareness

We first consider the effect of territorial awareness on species biodiversity. In general, it is well-known that, the spatial RPS game exhibits a transition of survival states from coexistence to extinction (which is presented by the uniform state) as individuals' mobility increases. The phase transition occurs when *M* exceed a certain value, referred to as a critical mobility $M_c = (4.5 \pm 0.5) \times 10^{-4}$, which is identified in Ref.^11^. To address the effect of territorial awareness, we consider two different mobility values $M=1 \times 10^{-5}$ and $M=1 \times 10^{-3}$ which eventually yield different survival states: coexistence and extinction, respectively, for different sensitivity parameter *k*.

**Figure 2:**
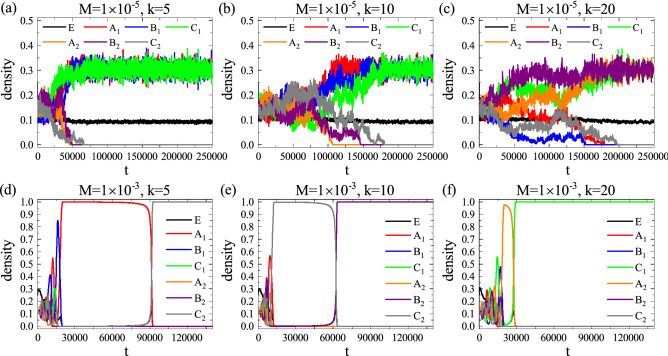
Time dependent evolution of densities in the system for different *M* and *k*. Top and bottom panels are obtained with $M=1\times 10^{-5}$ and $M=1\times 10^{-3}$, respectively, and the sensitivity parameter *k* is given by $k=5$, 10, and 20 from the left to right in each row. (**a**–**c**) Regardless of the choice *k*, the low mobility still leads species coexistence as usual. (**d**–**f**) At high mobility regimes, the system also always exhibit the extinction state.

In general, the total simulation time *T* in classic spatial RPS games is considered as $T = N$ which can yield the extinction for the critical mobility $M_c$^11^. In this regard, using the time $T=N$ may yield different results for species evolution and corresponding survival states due to stochastic events, and such behaviors may be induced by the choice of mobility. In our simulations, since we consider two different mobility values where the one (Fig. 2a–c) is quite lower and the other (Fig. 2d–f) is higher than $M_c$, we thus consider different simulation times at $M=1 \times 10^{-5}$ and $M=1 \times 10^{-3}$: more than 490, 000 and 180, 000 steps, respectively, to obtain robust features on species survival states. The time dependent evolution of densities are illustrated in Fig. 2 where the top and bottom panels are obtained from simulations with the first 250, 000 and 140, 000 steps, respectively.

Even if different *k* are considered, the panels in Fig. 2 show features similar to previous works^11,14–16,18,20^: coexistence and extinction for tops and bottoms, respectively. At the low mobility $M=1\times 10^{-5}$ as shown in Fig. 2a–c, even if the individuals located in different domains in each group disappear, the spatial RPS game eventually exhibits coexistence as *k* increases since some of individuals in *A*, *B*, *C* are survived. For instance, in our simulations, coexistence can be presented by survival of species $A_1$, $B_1$, $C_1$ (Fig. 2a–b) or $A_2$, $B_2$, $C_1$ (Fig. 2c). Since the typical waiting time for extinction is exponentially increasing to the size *N* at low mobility^11^, there will be extinction and eventually only one species will dominate the system after extremely long times. Thus, within the finite time steps, one type of each species will disappear slowly with the increase of *k* and the system exhibits coexistence.

On the other hand, the high mobility $M=1\times 10^{-3}$ leads the extinction and only one species dominate the whole domain. As shown in Fig. 2d–f, the extinction that is defined by the two types of the species disappear occurs and the only one species finally dominate the system [e.g., $C_2$, $B_2$, and $C_1$ for $k=5$, 10, and 20, respectively]. In this case, the increase in *k* has little effect on the disappearance of one of the species, but has a tendency to accelerate the complete extinction of the second species. Take Fig. 2d for example, when species $A_2$, $B_1$ and $B_2$ became extinct, $A_1$, $C_1$ and $C_2$ is left in the system, the density of $A_1$ in the system had an absolute advantage, while $C_1$ and $C_2$ had intraspecific interaction. Since the intensity of intraspecific interaction sensitive to territorial awareness was greater than that of interspecific interaction, the interaction was mainly intraspecific interaction between $C_1$ and $C_2$, then $C_1$ was defeated into extinction, $C_2$ preyed on the only specie $A_2$ remaining in the system and eventually occupied the whole system. As *k* value affects the intraspecific interaction intensity, it determines the waiting time for the extinction of two species in the system. For example, we found that the larger the *k* value is, the shorter the waiting time for the extinction of two species is, as shown in Fig. 2e–f. But this is only the observation result of a single simulation. Due to the randomness of the simulation, this phenomenon needs further verification, so we give specific results about the effect of *k* value on the average extinction time in the next section. Due to stochastic events during Monte-Carlo simulations, the combination of survival species for coexistence and extinction at the final step can be different, but the such states at two mobility regimes will be still maintained. Fig. 2 may impose the follows: territorial awareness on intraspecific interaction can eventually yield similar feature to previous works in a broad aspect, but the composition of the surviving species type for each state may vary.

### Average extinction time versus territorial awareness

While survival states in both cases are consistent with previous works on the effects of species migration in Fig. 2, we found an interesting feature that the evolutionary time when some type of species disappear is changed depending on *k*. To be concrete, at $M=1 \times 10^{-5}$, we found that one type of each species $A_1$, $B_1$, $C_1$ (Fig. 2a) will eventually coexist while their companion species $A_2$, $B_2$, $C_2$ are extinct as *t* exceeds $t \approx 50{,}000$. As *k* is increasing, the time point when one genus of each species disappears shows an increasing pattern as presented in Fig. 2b,c. The opposite trend can be captured at high mobility $M=1 \times 10^{-3}$, that is, the increase of *k* seems to shorten the evolution time of two species extinction in the system. Based on these observations, we may assume that the critical time for such disappearance phenomena has a certain relationship with *k* and the relation may differ to the choice of *M*.

**Figure 3:**
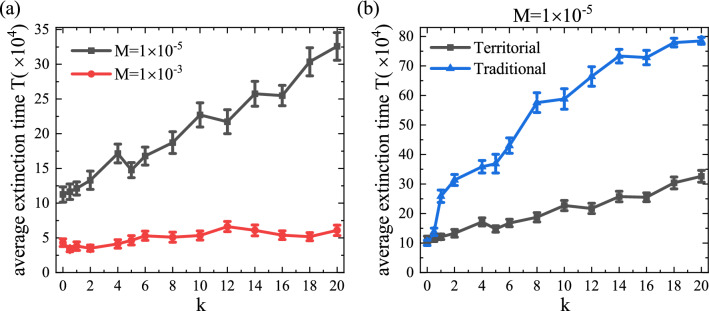
The average extinction time *T* as a function of the territorial sensitive parameter *k*. (**a**) Two cases of fixed mobility in territorial sensitive intraspecific interaction. For low mobility $M=1\times 10^{-5}$, the time *T* which is measured by detecting the time when one genus of each species disappears tends to increase with the increase of *k*, i.e., the high sensitivity of territorial awareness has the effect of delaying the waiting time for extinction. Similarly, it can be seen that at high mobility $M=1\times 10^{-3}$, an increase in *k* value will also delay the waiting time for extinction, but the effect is much more gentle. (**b**) At low mobility value $M=1\times 10^{-5}$, traditional intraspecific interaction (i.e., intraspecific interaction among all individuals of the same species, regardless of territorial residence) was compared with territorial sensitive intraspecific interaction. Here, for the traditional case, *k* represents intraspecific interaction intensity, and the running time of the simulation is 810, 000 steps. In the case that the final steady state has not occurred before the end of the simulation, we take the maximum time step ($t=810{,}000$) as the extinction time *T* value, which causes the blue line to become gentle when $k>14$. Compared with the traditional situation, the intraspecific interaction affected by territorial awareness significantly reduced the average extinction time, that is, accelerated the damage of species diversity in the system. The results were averaged from 30 independent simulations, and error bars (using standard errors, which defined as the sample standard deviation divided by the square root of the number of samples) are shown in the figure.

To answer the issue, we measure the average extinction time *T*. In classic RPS games, traditionally, the extinction state on spatially extended systems has been identified by the uniform state that only one species dominates whole domain^11,14–16,18,20^. As shown in Fig. 2a–c which ultimately describe a coexistence state in a finite time, however, any one of type in each species disappeared and the time associated with the phenomena is changed by the strength of *k*. In a slightly different aspect to the classic meaning of extinction, we here define the average extinction time *T* with respect to the regime of mobility: (a) the evolutionary time when one genus of each species disappears for low mobility and (b) the time when two of the three species disappear completely for high mobility. In this consideration, for both given cases of *M* in Fig. 2, the average extinction time *T* in each *k* is measured from 30 independent realizations and presented in Fig. 3.

As shown in Fig. 3a, we find clearly that the average extinction time is obviously affected by the strength of sensitivity coefficient *k*, especially, when the mobility is low. When species has no consciousness on territories ($k=0$), the system becomes exactly the classic RPS model^11^ since intraspecific interaction is undefined, and the waiting time *T* generally tends to increase exponentially to the choice of *M*. However, our simulation shows the *T* is approximately measured at $T=110{,}000$ at $k=0$. Traditionally, it is well known that the average waiting time for extinction in the classic RPS game is taken $T=N$ near the critical mobility regime ($M \approx M_c$), and the coexistence duration is exponentially increasing as *M* decreases from $M_c$. Within this knowledge, our simulation results may seem inconsistent with the general concept of extinction time. In our model, however, the definition of extinction is different at the low mobility regime, and the change into a single RPS system as one genus of each individual disappears may have a similar meaning to the previous definition of extinction in some sense, the above result can be said to be reasonable.

The important point is actually addressed for $k>0$. In this case, species can allow intraspecific interaction and the strength of intraspecific interaction is also increasing since the territorial awareness is intensified. As a result, it is found that the average extinction time *T* shows a tendency to gradually increase with the increase of *k* at $M=1 \times 10^{-5}$. In addition, this trend can also be observed at $M = 1 \times 10^{-3}$, but it is more gradual. To investigate whether the tendency to prolong the waiting time for extinction time at low migration rates is caused by territorial awareness or the existence of intraspecific interaction, we compared traditional intraspecific interaction (i.e., intraspecific interaction among all individuals of the same species, regardless of territorial residence, which equivalent to removing the condition $i\ne j$ from Relation (2)) with territorial-sensitive intraspecific interaction in our model, the results are shown in Fig. 3b. We found that in the presence of intraspecific interaction, the average extinction time increased with the intensity of intraspecific interaction. Specifically, the stronger the intraspecific interaction, the slower the loss of species diversity. However, compared with the traditional situation, intraspecific interaction influenced by territorial consciousness controlled the delay of extinction to a certain extent. Even if our simulations have been carried on for two specific *M*, it is obvious that the territorial awareness can affect the average extinction time, and we suggest that a strong sense of territoriality can also delay species extinction and lead to long-term coexistence of systems at low mobility regimes, although the introduction of territoriality leads to faster damage to species diversity than is traditionally the case, while it does not affect significantly on the extinction time and the biodiversity (which eventually appears as extinction) at high mobility regimes.

### Evolution of the interface between territories

**Figure 4:**
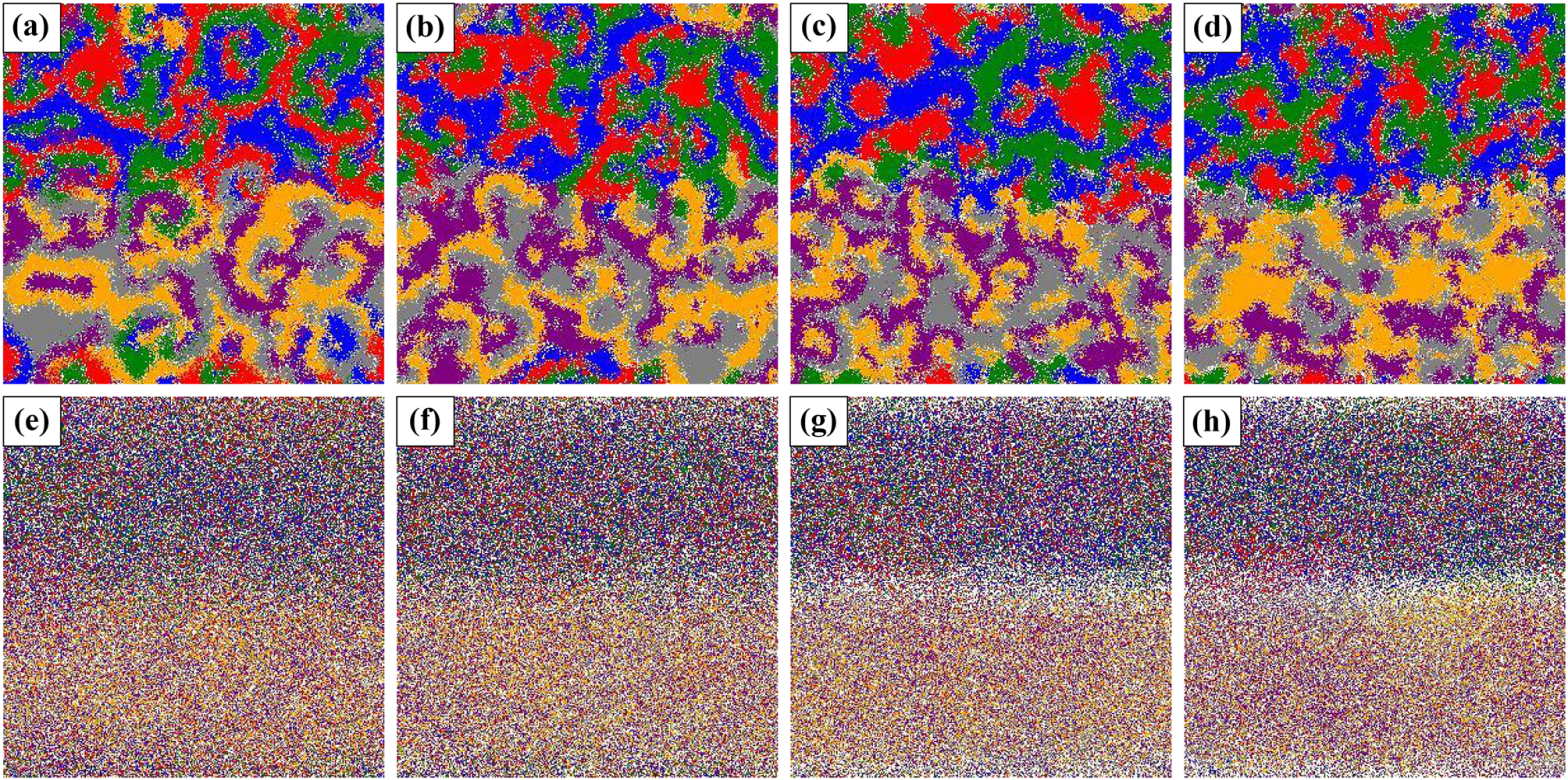
The typical snapshots of evolution on patterns at $t=1000$ for different *k*: 0.5 for (**a**) and (**e**), 2 for (**b**) and (**f**), 10 for (**c**) and (**g**), and 20 for (**d**) and (**h**), where the mobility is considered as $M = 1\times 10^{-5}$ for tops and $M=1 \times 10^{-3}$ for bottoms. Different colors correspond to different species types, as shown in Figs. 1 and 2, with white indicating vacancy. As *k* increases, the invasion among species between two territories occurs more gradually, and such phenomena are clearly observed for the high mobility as shown in the panel (**h**).

From the investigation on the average extinction time in Fig. 3, we know that the territorial awareness can affect not only species survival but also the maintenance period of survival states. Here, we may wonder why the territorial awareness can affect the waiting time to extinction. In order to investigate such an issue, we observe evolution of the spatial system, in particular invasion between species near the border on two territories, i.e., *the evolution of the interface*. To capture the phenomena in detail, we consider pattern formations associated with the given two mobility values at the initial state of the evolution (e.g. $t=1000$) which are represented in Fig. 4.

The top and bottom panels in Fig. 4 exhibit spatial patterns for the low and high mobility values, respectively. For $M=1 \times 10^{-5}$, when the value *k* is small such as $k=0.5$ (see Fig. 4a), interspecific interaction can occur more frequently than intraspecific interaction among all pairwise reactions (1)–(4). The system can exhibit similar pattern formations to the classic RPS game^11^. Three species, even if they are distinguished into six subgroups, are spirally entangled with clearly exhibiting spiral waves which are appeared in both two territories. Since the given lattice has periodic boundaries, species in both territories can migrate to the other region each other, but such migration is weak because the normalized probability for migration (Relation (4)) is small at the low mobility. Thus, when the system exhibits coexistence, it may be possible to predict that the top and bottom territories present dominance of species $X_1$ and $X_2$ ($X \in \{A, B, C\}$), respectively, while our simulations only present spatial patterns at the first 1,000 steps which may be too short to lead phase transitions.

We also find that the spiral-wave patterns are getting to fuzzy as *k* increases. In particular, such fuzzy patterns are conspicuous near the boundary between the two territories at the large *k* (see Fig. 4c,d). The increase of *k* directly means the intensification of intraspecific interaction, and according to the setting on the initial distribution of population, intraspecific interaction will have many chances to occur in the vicinity of the boundary than near the top and bottom periodic boundaries. Frequent intraspecific interaction can provide as many chances to allow reproduction as possible, and high intraspecific interaction rate can dominate on pairwise invasions than interspecific interaction.

In the vicinity of the border between two territories, the occurrence of intraspecific interaction is observed more prominently at $M=1 \times 10^{-3}$, and such features are clear as *k* increases. To be concrete, compared with figures among Fig. 4e–h, we found that empty sites are produced near the border and their presence is clear for high strength *k* such as 10 and 20 (Fig. 4g–h). In this case, the two domains appear to be more clearly divided and each domain is dominated by a single RPS system. Each single RPS system shows extinction state (only one genus survives) at high mobility, and eventually shows extinction state through interspecific or intraspecific interaction depending on the type of surviving genus. This is in good agreement with the results we obtained in Fig. 2. However, it can be seen from Fig. 3 that the time for each domain system to reach extinction at high mobility is very short compared to that at low mobility, and this has no relation with the degree of territorial awareness in interspecific interaction.

**Figure 5:**
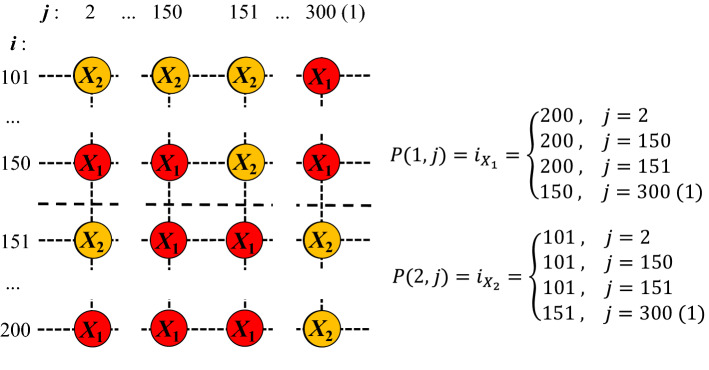
Diagram of the definition and numerical determination of *P*(1, *j*) and *P*(2, *j*). In accordance with the definition in Fig. 1a, species are distributed in two different fields on the network. Here, $X_{1}\,(X_{2},\,X\in A,B,C)$ is used to represent individual species initially distributed in the upper (lower) half of the territory, and colored nodes respectively represent their distribution positions in the lattice network. Each node in the network is given a position coordinate, defined by its row, column number *i* and *j*. In the diagram, for each column *j*, we can see two scenarios: the species remaining in its territory (when $j=300(1)$, with (1) represents periodic boundary), and the species invading rival territory (when $j=2,\,150,\,151$). In accordance with the descriptive definition of $P(1,j)\,(P(2,j))$ in formula (5), we take the position of $X_{1}\,(X_{2})$ closest to the two neighborhood interfaces as the value of $P(1,j)\,(P(2,j))$ for the first case, while for the second case, we take the farthest point in the opponent’s domain where $X_{1}\,(X_{2})$ can invade as the value of $P(1,j)\,(P(2,j))$. The right hand side of the diagram shows the corresponding values.

From our simulations, we find that: the relationship between territorial awareness and the average extinction time is particularly prominent at the low mobility, and the likelihood of intraspecific interaction is relatively high near territorial boundaries. Under these considerations, we may expect a new relationship between the delay of the extinction time and boundary of two territories. To uncover this veil, we try to quantify a width for occurrence of intraspecific interaction near the border between two area with respect to the territorial awareness. Specifically, we give each node on a two-dimensional grid a coordinate, defined by its row and column position. For each column $j=1,\ldots ,L$, calculate the interface width, defined as *I*:

5 $$\begin{aligned} I_{j}= {\left\{ \begin{array}{ll} P(1,j)-P(2,j),&{}\quad \text {if } P(1,j)>P(2,j)\\ 0,&{}\quad \text {if } P(1,j)<P(2,j) \end{array}\right. } \end{aligned}$$

with the quantity *P*(1, *j*) is the abscissa of the individual of species $X_{1}\,(X\in A,B,C)$ in column *j* reaching the position nearest to the interface in its own region when any individual of species $X_1$ does not exist in the territory of species $X_2$, or furthest from the interface in its opponent region when one or more individuals of species $X_1$ invade the territory of species $X_2$. *P*(2, *j*) is similarly defined, corresponding to individuals of species $X_{2}\,(X\in A,B,C)$. In order to avoid the effect of periodic boundary conditions, we only consider 100 row positions in the middle of the given lattice, i.e., $100<P(1,j),\,P(2,j)\le 200$. The specific definition and numerical determination of *P*(1, *j*) and *P*(2, *j*) are illustrated in Fig. 5.

**Figure 6:**
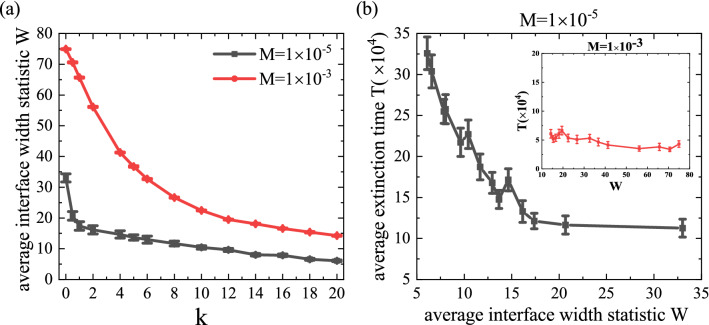
The evolution of interface width statistics at time step $t=1000$. (**a**) The average interface width statistic *W* as a function of *k* for two fixed mobility values. For two mobility values, we have a common feature that the value *W* is decreasing as *k* increases. In this case, the decrease in *W* shows a sharp change at the low *M* compared to the case of high *M*. Such a decrease shows a gradual change in the value of *k* above a certain level, and the decrease in *W* means that intraspecific interaction according to territorial awareness is eventually limited to the vicinity of the boundary between two domains. (**b**) Relationship between mean extinction time *T* and interface width statistic *W* for two fixed mobility values (see the illustration for $M=1 \times 10^{-3}$). The relevant values correspond to the results shown in Figs. 3a, 6a with different values of *k*. The mean extinction time decreases with the interface width, which is more obvious at low migration rate and more gentle at high migration rate. The results were averaged from 30 independent simulations, and error bars (using standard errors, which defined as the sample standard deviation divided by the square root of the number of samples) are shown in the figure.

By averaging the interface width of each column, the interface width statistics of the whole system can be obtained:

6 $$\begin{aligned} W=\frac{\sum _{j=1}^{L} I_{j}}{L}, \end{aligned}$$

by this definition, *W* can be understood as the average range at which species in two territories invade each other near the interface. At a particular time step, we can calculate the values of *W* for two mobility values $M=1 \times 10^{-5}$ and $1 \times 10^{-3}$. Here, we pay attention to the evolution of the initial stage, take time step $t=1000$, and calculate the average value from 30 independent realizations, the results are shown in Fig. 6.

Figure 6a shows the dependence of the average interface width statistic *W* on the strength of sensitivity to the territory *k*. For $M=1 \times 10^{-5}$, the average interface width statistic *W* is decreasing as *k* increases, which means interactions among species on two different regions can only occur near the border of two area. In the rest of the area except near the border, species may have many chances to do reactions except intraspecific interaction since only species $X_1$ or $X_2$ ($X \in \{A,B,C\}$) can interact by itself in the rest area due to the rare event of migration at the low mobility regime, which can validate the long-term coexistence of species $X_1$ and $X_2$ ($X \in \{A,B,C\}$) in each rest area. Such a decreasing phenomenon is also obtained at the high mobility $M=1 \times 10^{-3}$ even if the decrease is lower than in the case of $M=1 \times 10^{-5}$. Thus, in the vicinity of the border between two regions, intraspecific interaction can occur frequently with high *k* and invasion, i.e., interspecific interaction, among different species groups is slowing down which eventually is consistent with Fig. 4. In addition, in order to illustrate the effective measurement of interface width statistics for the speed of system evolution, we describe the relationship between the average extinction time *T* and interface width statistics *W* in Fig. 6b by combining the results obtained in Fig. 3a. It can be seen from the figure that *T* decreases with *W* under both mobility conditions, but the trend of high mobility is not as obvious as that of low mobility. This declining trend illustrates the fact that at the same time step, the wider the interface between two regions, the faster the system evolves and reaches what we define as an extinction state. In short, larger values of *k* result in smaller statistics indicating slower invasion another territory of the species.

## Discussion

In the existing studies on intraspecific interaction in RPS games, members of each species are generally on equal footing, and in most cases, the intraspecific interaction that affects coexistence or extinction maintains intrinsic symmetry. In other words, it has been assumed that interaction within the same group occurs at a certain level regardless of the circumstances of the members. However, intraspecific interaction is highly likely to occur as a conflict caused by the situation in which each member is actually placed, and one of the reasons that can cause such conflict is the territory. Therefore, as an individual recognizes a territory, the question of how the frequency of interaction changes and how it affects species diversity is a natural phenomenon.

As a matter of fact, territorialism that the consciousness of individuals or groups to fight for new territory or defend their area from encroachment by others is a common feature in nature. In this regard, the main contribution of our work is the role of territorial awareness that closely affects the strength of intraspecific interaction. To accomplish this goal, we study the spatial RPS game on a square lattice, subject to territorial awareness dependent intraspecific interaction between individuals in each same group which will be placed on two different area.

Through extensive numerical simulations, we found that, at high mobility regime, the system would exhibit the extinction state regardless of the strength of territorial awareness, which is same to the classic model. Under the low mobility that results in species coexistence, however, the increase of territorial awareness can play a significant role to affect intraspecific interaction, and eventually yields the delay of extinction time which is measured by the time when one of genus in each species disappears. In other words, the stronger territorial awareness leads the stronger intraspecific interaction and increases the coexistence time. Such phenomena is accompanied with the slowing down of invasion near the interface between two area, and exploiting a quantity for interface width validated the numerical findings. Compared with previous studies on intraspecific interaction in spatial RPS games, our results suggest that intraspecific interaction with territorial considerations follows the results of traditional intraspecific interaction, prolonging the coexistence time of all species, which is also likely to benefit species diversity, but this particular form of interaction is less effective in maintaining species diversity than traditional interaction. Our results also enrich foundations to understand evolution of species in RPS games.

## Methods

Our simulations were carried out in a two-dimensional square lattice of size $N = L\times L$ with periodic boundaries. Each grid contains one individual and individuals of three species or vacancies are randomly placed in each grid as the initial state of the system. The interaction between individuals is simulated by Monte Carlo (MC) method. At each time step, a randomly selected individual interacts with a randomly selected neighbor node. For convenient to make unbiased comparison with some previous works on intraspecific interaction, we set $p_{1} = p_{2} = r =1, k_{A}=k_{B}=k_{C}=k$ in this work. For a pair of adjacent nodes, interspecific interaction, intraspecific interaction, reproduction, and migration were conducted with probabilities: $p_{1}/(p_{1} + k \cdot p_{2} + r + m)$, $k \cdot p_{2}/(p_{1} + k \cdot p_{2} + r + m)$, $r /(p_{1} + k \cdot p_{2} + r + m)$, and $m/(p_{1} + k \cdot p_{2} + r + m)$, so the rates of interaction, reproduction, and migration are normalized. The success of the interaction depends on the state of the two nodes, for example, if reproduction is selected, but there is no vacancy site, the reaction fails. We aimed to study the dynamics of species diversity changes caused by species mobility rate and territorial awareness by changing the mobility rate *M* and sensitive coefficient *k*.

## References

[1] May RM (2019). Stability and Complexity in Model Ecosystems.

[2] Szabó G, Fath G (2007). Evolutionary games on graphs. Phys. Rep..

[3] Perc M, Szolnoki A (2007). Noise-guided evolution within cyclical interactions. N. J. Phys..

[4] Lai Y-C, Liu Y-R (2005). Noise promotes species diversity in nature. Phys. Rev. Lett..

[5] Sinervo B, Lively CM (1996). The rock-paper-scissors game and the evolution of alternative male strategies. Nature.

[6] Kirkup BC, Riley MA (2004). Antibiotic-mediated antagonism leads to a bacterial game of rock-paper-scissors in vivo. Nature.

[7] Neumann GF, Jetschke G (2010). Evolutionary classification of toxin mediated interactions in microorganisms. BioSystems.

[8] Nahum JR, Harding BN, Kerr B (2011). Evolution of restraint in a structured rock-paper-scissors community. Proc. Natl. Acad. Sci..

[9] Hofbauer J, Sigmund K (1998). Evolutionary Games and Population Dynamics.

[10] Kerr B, Riley MA, Feldman MW, Bohannan BJ (2002). Local dispersal promotes biodiversity in a real-life game of rock-paper-scissors. Nature.

[11] Reichenbach T, Mobilia M, Frey E (2007). Mobility promotes and jeopardizes biodiversity in rock-paper-scissors games. Nature.

[12] Toupo DF, Strogatz SH (2015). Nonlinear dynamics of the rock-paper-scissors game with mutations. Phys. Rev. E.

[13] Ni X, Wang W-X, Lai Y-C, Grebogi C (2010). Cyclic competition of mobile species on continuous space: pattern formation and coexistence. Phys. Rev. E.

[14] Shi H, Wang W-X, Yang R, Lai Y-C (2010). Basins of attraction for species extinction and coexistence in spatial rock-paper-scissors games. Phys. Rev. E.

[15] Ni X, Yang R, Wang W-X, Lai Y-C, Grebogi C (2010). Basins of coexistence and extinction in spatially extended ecosystems of cyclically competing species. Chaos.

[16] Kim B, Park J (2017). Basins of distinct asymptotic states in the cyclically competing mobile five species game. Chaos.

[17] Müller APO, Gallas JAC (2010). How community size affects survival chances in cyclic competition games that microorganisms play. Phys. Rev. E.

[18] Park J, Jang B (2021). Structural stability of coexistence in evolutionary dynamics of cyclic competition. Appl. Math. Comput..

[19] Yang R, Wang W-X, Lai Y-C, Grebogi C (2010). Role of intraspecific competition in the coexistence of mobile populations in spatially extended ecosystems. Chaos.

[20] Park J, Do Y, Jang B, Lai Y-C (2017). Emergence of unusual coexistence states in cyclic game systems. Sci. Rep..

[21] Park J, Do Y, Jang B (2018). Multistability in the cyclic competition system. Chaos.

[22] Park J (2018). Balancedness among competitions for biodiversity in the cyclic structured three species system. Appl. Math. Comput..

[23] Park J, Do Y, Huang Z-G, Lai Y-C (2013). Persistent coexistence of cyclically competing species in spatially extended ecosystems. Chaos.

[24] Park J (2019). Fitness-based mutation in the spatial rock-paper-scissors game: Shifting of critical mobility for extinction. EPL.

[25] Han X, Chen B, Hui C (2016). Symmetry breaking in cyclic competition by niche construction. Appl. Math. Comput..

[26] Svanbäck R, Bolnick DI (2007). Intraspecific competition drives increased resource use diversity within a natural population. Proc. R. Soc. B Biol. Sci..

[27] Ward AJ, Webster MM, Hart PJ (2006). Intraspecific food competition in fishes. Fish Fish..

[28] Wang X-G, Messing RH (2003). Intra-and interspecific competition by *Fopius arisanus* and *Diachasmimorpha tryoni* (Hymenoptera: Braconidae), parasitoids of tephritid fruit flies. Biol. Control.

[29] Chase JM (2002). The interaction between predation and competition: A review and synthesis. Ecol. Lett..

[30] Chesson P, Kuang JJ (2008). The interaction between predation and competition. Nature.

[31] Lorenz K (2002). On Aggression.

[32] Tinbergen N (1957). The functions of territory. Bird Study.

[33] Tinbergen N (2020). The Study of Instinct.

[34] Mosser A, Packer C (2009). Group territoriality and the benefits of sociality in the African lion, *Panthera leo*. Anim. Behav..

[35] RASA OE (1987). The dwarf mongoose: A study of behavior and social structure in relation to ecology in a small, social carnivore. Adv. Study Behav..

[36] Henschel JR, Skinner JD (1991). Territorial behaviour by a clan of spotted hyaenas *Crocuta crocuta*. Ethology.

[37] Boydston EE, Morelli TL, Holekamp KE (2001). Sex differences in territorial behavior exhibited by the spotted hyena (Hyaenidae, *Crocuta crocuta*). Ethology.

[38] Redner S (2001). A Guide to First-Passage Processes.

